# Allogeneic hematopoietic stem cell transplantation and pre-transplant strategies in patients with NPM1-mutated acute myeloid leukemia: a single center experience

**DOI:** 10.1038/s41598-023-38037-5

**Published:** 2023-07-04

**Authors:** Paul Jäger, Christina Rautenberg, Jennifer Kaivers, Annika Kasprzak, Stefanie Geyh, Ben-Niklas Baermann, Rainer Haas, Ulrich Germing, Thomas Schroeder, Guido Kobbe

**Affiliations:** 1 Department of Hematology, Oncology and Clinical Immunology, Heinrich-Heine-University, Medical Faculty, Moorenstr. 5, 40225 Düsseldorf, Germany; 2 Department of Hematology and Stem Cell Transplantation, University Hospital Essen, Essen, Germany

**Keywords:** Acute myeloid leukaemia, Chemotherapy

## Abstract

Patients with acute myeloid leukemia (AML) and nucleophosmin 1 gene mutations (NPM1^mut^) show a favorable prognosis with chemotherapy (CT) in the absence of negative prognostic genetic abnormalities. Between 2008 and 2021 64 patients with NPM1^mut^AML received alloHSCT because of additional adverse prognostic factors (1st line), inadequate response to or relapse during or after CT (2nd line). To expand the evidence in alloTX in NPM1^mut^ AML, clinical and molecular data were retrospectively analyzed with respect to pre-transplant strategies and outcome. Patients with minimal residual disease negative (MRD−) CR at transplant had better 2-y-PFS and 2-y-OS (77% and 88%) than patients with minimal residual disease positive (MRD+) CR (41% and 71%) or patients with active disease (AD) at transplant (20% and 52%). The 2nd line patients with relapse after completing CT responded well to high dose cytarabine based salvage chemotherapy (salvage CT) in contrast to patients relapsing while still on CT (90% vs 20%, P = 0.0170). 2-y-PFS and 2-y-OS was 86% in patients who achieved a 2nd MRD− CR pre alloHSCT. Outcome in NPM1^mut^AML depends on disease burden at alloHSCT. Time and type of relapse in relation to CT are predictive for response to salvage CT.

## Introduction

Treatment decisions in patients with acute myeloid leukemia (AML), particularly concerning the use of allogeneic hematopoietic stem cell transplantation (alloHSCT), are mainly influenced by disease biology and patients’ characteristics such as performance status and eligibility for intensive therapy. Disease biology is characterized by the presence of genetic and molecular alterations, which can be classified with regard to their prognostic impact according to the European LeukemiaNet (ELN) classification^1,2^. Patients with isolated nucleophosmin 1 gene mutated (NPM1^mut^) AML are considered to convey a favorable prognosis^3–8^. Additionally, the detection of the NPM1^mut^ gene by reverse transcription polymerase chain reaction (RT-PCR) represents a reliable marker to track measurable residual disease (MRD), offering the opportunity to sensitively monitor the course of disease on the submicroscopic level during conventional chemotherapy (CT) as well as after alloHSCT and to guide early therapeutic interventions^9–12^. However, patients with NPM1^mut^ AML and additional FMS-like tyrosine kinase 3 internal tandem duplication especially with a ratio > 0.5 (FLT3-ITD^high^) or adverse structural chromosomal aberrations have a poor outcome with CT and are widely considered as candidates for alloHSCT in first complete remission (1st CR, 1st line patients)^5,8^. Furthermore, a relevant proportion of patients without additional adverse prognostic factors relapse after front-line CT. AlloHSCT as second line therapy (2nd line patients) can facilitate long-term disease control in some of these patients. Relapse may be detected early on a molecular level (MRD+) or as frank hematological relapse after or during front-line CT^6,9,10,13^. Currently, it is unknown whether patients in relapse require immediate therapy before alloHSCT with the intention to achieve a second CR. Recent retrospective studies suggest that achieving a second, MRD negative (MRD−) CR before transplant is predictive for long term remission after transplant^13–16^. Likewise, the remission status prior to alloHSCT also may have an impact on the choice of the conditioning regimen before alloHSCT as the benefit of more intensive conditioning to reduce or eliminate disease burden in MRD+ patients or patients with AD has been shown to be controversial^16–22^. Thus, the optimal peri-transplant management of this specific patient group has not been addressed in detail so far.

Still, only a few reports covering a total of 256 individuals have specifically analyzed the impact of alloHSCT in patients with NPM1^mut^ AML so far^13–16^.

To augment and to expand the evidence we analyzed the impact of alloHSCT and peri-transplant strategies in a well-annotated single center cohort of 64 NPM1^mut^ AML patients treated at our center from 2008 to 2021.

## Patients and methods

### Patients

**Figure 1:**
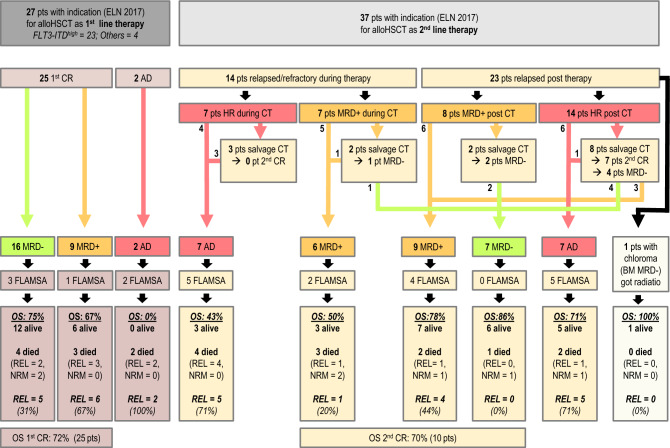
CONSORT diagram: *HR* hematological relapsed or refractory disease with morphological blast detection in BM > 5%, *CT* conventional (front-line) chemotherapy, *CR* morphological complete remission, *FLAMSA* fludarabine, amsacrine and cytarabine based sequential conditioning regimen, *salvage CT* high-dose cytarabine based salvage chemotherapy, *alloHSCT* allogeneic hematopoietic stem cell transplantation, *MRD+* measurable residual disease, *MRD−* no measurable residual disease, *NRM* non-relapse mortality, *REL* relapse, *OS*: overall survival, *AD* active disease, *pts* patients. See also supplemental text to this figure.

Between 2008 and 2021, 64 patients with NPM1^mut^ AML at our institution had an indication for alloHSCT either as part of their first-line therapy (n = 27, 42%) due to ELN 2017 intermediate or poor-risk genetic features or as salvage therapy in case of molecular persistence/relapse or hematologic relapse in those initially classified as favorable risk (n = 37, 58%) (Table 1; Fig. 1). Details of the treatment given before alloHSCT in the 1st line patients are given in Table S1 and the treatment given as salvage for envisaged alloHSCT for 2nd line patients is found in Table S2.

**Table 1:** Patient and transplant characteristics NPM1^mut^ AML.

Characteristic	n	%
Number of patients	64	100
Median age (range), years	51 (32–70)	
Gender		
Female	41	64
Male	23	36
Karyotype		
Normal	53	83
Abnormal	11	17
Molecular genetics		
FLT3-ITD^high^	23	36
FLT3-ITD^low^	6	9
FLT3-TKD	5	9
Genetic risk according ELN 2017		
Favorable	37	58
Intermediate	26	41
Adverse	1	2
Transplant characteristics		
HLA-matching		
Matched related donor	14	22
Matched unrelated	41	64
Mismatched unrelated	9	14
ATG		
Yes	48	75
No	16	25
Conditioning regimen		
Sequential FLAMSA based	22	34
Other	42	67

*AML* acute myeloid leukemia, *alloHSCT* allogeneic hematopoietic stem cell transplantation, *FLAMSA* fludarabine, amsacrine and high dose cytarabine based sequential conditioning regime.

### Molecular genetic analysis

Screening for NPM1 and FLT3 mutations was performed in patients with newly diagnosed AML according to accredited methods of different laboratories. Serial samples for quantitative MRD monitoring by RT-PCR of NPM1 mutations were obtained during the course of treatment. Bone marrow aspirates (BM) were requested on regeneration after each cycle of chemotherapy, and then at least every 3 months. For patients receiving alloHSCT, samples were regularly requested before alloHSCT, at d28, at d60, at d100, at d200, at d300 and then at 3-month intervals for at least 2 years. Thereafter, blood samples continued to be analyzed for the reappearance of NPM1 MRD at 3- to 6-month intervals. In case of molecular relapse or if abnormalities in the blood count occurred, a bone marrow aspiration was performed again. These close-meshed and intensive remission controls are part of the standard monitoring at our center and were identical in all cohorts. The results from MRD analyses were available to the transplant team.

### Relapse definition

Consistent with ELN guidelines, molecular relapse was defined as either conversion of MRD negativity to MRD positivity detected by RT-PCR or increase of MRD ≥ 1 log_10_ between any two positive samples measured in the same tissue (PB or BM) in patients with MRD at low level. Conversion from negative to positive MRD in PB or BM was confirmed within 4 weeks, in a second consecutive sample with bone marrow aspiration^23^. Morphological relapse/persistence was defined as reoccurrence or persistence of blasts > 5% in bone marrow or reoccurrence or persistence of blasts in peripheral blood.

Relapse post therapy was defined according to aforementioned criteria following reconstitution after the last completed consolidation cycle and relapse during therapy was defined according to aforementioned criteria after reconstitution in later consolidation cycles. Details are given in Table S2.

### Statistical analyses

Overall survival (OS) following transplant was calculated as the time from alloHSCT to death from any cause or last follow-up in survivors. Progression free survival (PFS) was defined as the time from alloHSCT until progression to molecular or hematologic relapse or death with those censored at last contact who were alive and had not experienced molecular or hematologic relapse until then. Cumulative incidence of relapse (CIR) and non-relapse mortality (NRM) were calculated using cumulative incidence (CI) estimates and considered as competing risks. All time-to-event curves were estimated using the Kaplan–Meier method and log-rank test for univariate comparisons. For all analyses, a P value < 0.05 was considered to be statistically significant. Statistical analyses were performed using GraphPad Prism^®^ 5.01 (GraphPad Software Inc., La Jolla, USA) and SPSS Statistic for Windows (SPSS Inc. Chicago, IL) and further details are given in the respective figure legend.

### Ethical approval

The retrospective analysis was approved of the Ethics Committee at the Faculty of Medicine of Heinrich Heine University Düsseldorf (approval number: 2022-2047). After review by the Ethics Committee at the Faculty of Medicine of Heinrich Heine University Düsseldorf, no informed consent is required for this retrospective data analysis. All methods were performed in accordance with the relevant guidelines and regulations.

### Conference presentation

Parts were presented at the 48th Annual Meeting of the European Society for Blood and Marrow Transplantation (EBMT 2022) in Prague, Czech Republic from the 19th to the 23rd of March 2022 as poster presentation (AS-EBMT-2022-01092).

## Results

### Overall cohort analysis

**Figure 2:**
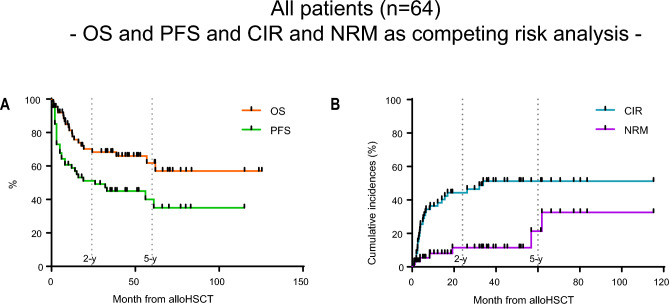
(**A**) Kaplan–Meier survival curve with overall survival (OS) and progression free survival (PFS) month from alloHSCT of all patients. (**B**) Cumulative incidence of relapse (CIR) and non-relapse mortality (NRM) as competing risks analysis month from alloHSCT of all patients.

In total, 64 patients with NPM1^mut^ AML had an indication for alloHSCT and received alloHSCT at our institution since 2008. In these 64 patients the median follow-up was 2.7 years from transplant (range 0.1–10.4 years). Two-year-progression free survival (2-y-PFS) and the 2-year-overall survival (2-y-OS) of the entire cohort was 53% and 72%, respectively (Fig. 2A).

In total, 28 patients (44%) relapsed after alloHSCT. Twenty-one patients (33%) died after alloHSCT. The cause of death was disease relapse in 14 patients (22%) and in seven patients (11%) death was not attributed to relapse. CIR and NRM were considered as competing risk (Fig. 2B). For more detailed information on the different subgroups, please see the supplemental text to Fig. 1.

### Survival according to pre-transplant remission and molecular MRD status

Among all patients who received alloHSCT, 23 patients (36%) had MRD− and 24 patients (38%) had MRD+ CR before alloHSCT and 16 patients (25%) were transplanted with active disease (AD). One patient with chloroma pre alloHSCT was excluded from further analysis.

**Figure 3:**
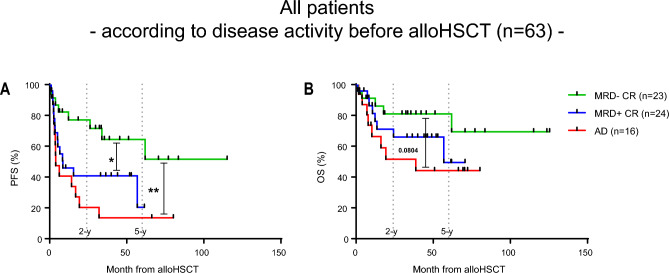
Kaplan–Meier survival curves. (**A**) Progression free survival (PFS) and (**B**) overall survival (OS) month from alloHSCT of all patients according to pre alloHSCT remission status. Log-rank (Mantel-Cox) test was used to test for statistical significance in survival curves. Statistical significance was established at asterisks displaying P values: *P < 0.05, **P < 0.01.

Patients with MRD− CR at transplant had a significantly higher 2-y-PFS and (ns) 2-y-OS (77% and 81%) than patients with MRD+ CR (41% and 71%) or patients with AD at transplant (20% and 52%) (PFS: MRD− CR vs MRD+ CR, P = 0.0118; MRD− CR vs AD, P = 0.0018) (Fig. 3A,B).

In the first-line situation, we observed a higher 2-y-PFS of 73% in MRD− CR compared to 33% (ns) when patients had MRD+ CR before alloHSCT, but there was no relevant difference concerning 2-y-OS (Figure S1A,B). Two patients with 1st line indication had AD at transplant and died of relapse 10 and 39 months after alloHSCT.

**Figure 4:**
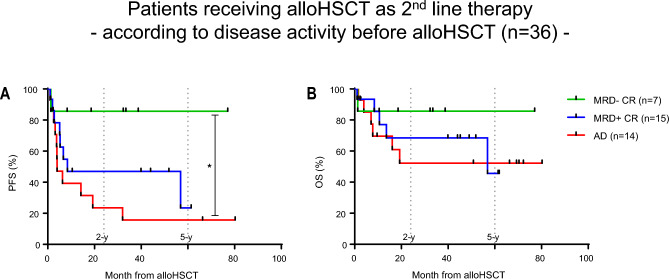
Kaplan–Meier survival curves. (**A**) Progression free survival and (**B**) overall survival month from alloHSCT of patients with 2nd line indication for alloHSCT according to pre alloHSCT remission status. Log-rank (Mantel-Cox) test was used to test for statistical significance in survival curves. Statistical significance was established at asterisks displaying P values: *P < 0.05.

For 2nd line patients the 2-y-PFS and 2-y-OS was 86% when MRD− CR at transplant was achieved through salvage CT. If MRD was persistently detectable in patients with morphological CR, 2-y-PFS and 2-y-OS was 47% and 68%, respectively. When AD was present at alloHSCT, 2-y-PFS and 2-y-OS was 24% and 52%, respectively (PFS: MRD− CR vs AD, P = 0.0165) (Fig. 4A,B).

### Pre-transplant strategies in NPM1^mut^ AML

MRD− CR was achieved in 16 of 27 patients (59%) with 1st line indication for alloHSCT (Supplement Table 1). Nine patients exhibited MRD+ CR and two patients were primary refractory. Only eight patients with concomitant FLT3-ITD^high^ had additional treatment with midostaurine but without being able to show a significant result given the small cohort size. Details are shown in Table 1 of the Supplement.

**Figure 5:**
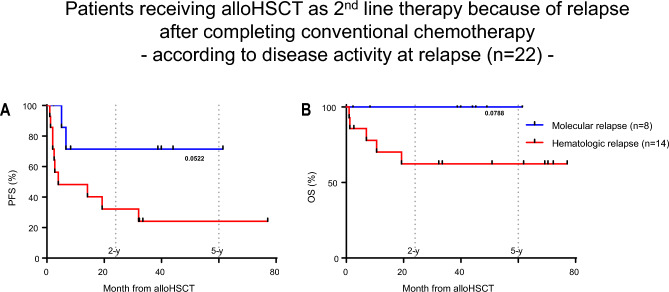
Kaplan–Meier survival curves. (**A**) progression free survival (PFS) and (**B**) overall survival (OS) month from alloHSCT of patients with 2nd line indication for alloHSCT because of relapse post conventional front-line chemotherapy according to relapse characteristics. Log-rank (Mantel-Cox) test was used to test for statistical significance in survival curves. P values are shown.

In patients where the transplant indication was triggered by relapse or persistent disease during or after CT (2nd line indication, n = 37, 58%), 2-y-PFS and 2-y-OS after alloHSCT was best with 71% and 100%, respectively, when relapse was detected on a molecular level after completion of CT (Fig. 5A,B). Overall, relapse after completion of therapy compared to patients relapsing during CT (data not shown) and relapse detected on the molecular level was associated with better progression free survival (Figure S2A,B).

**Figure 6:**
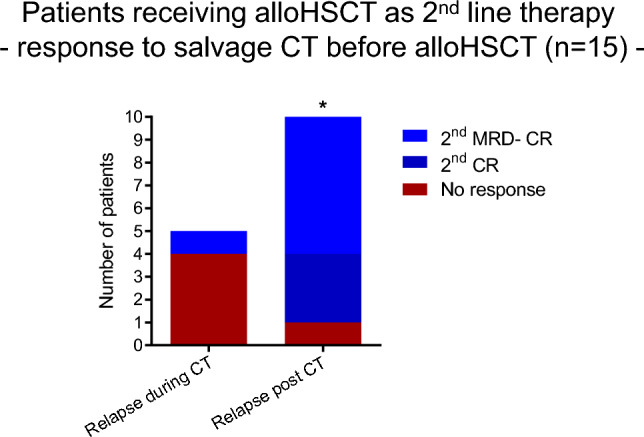
Number of patients with 2nd line indication for allo HSCT responding to S-CT post relapse depending on relapse time. Fisher's exact test was used to test for statistical significance. Statistical significance was established at asterisks displaying P values: *P < 0.05.

Fifteen patients received high-dose cytarabine based salvage chemotherapy (salvage CT: FLAG-Ida, n = 11; HAM, n = 2; FLAMSA, n = 1; HD AraC + Thiotepa, n = 1) before alloHSCT, starting in median 21 days from relapse (range 16–130 days) with an overall response rate of 67%. Patients with molecular or hematologic relapse after completing CT responded well to salvage CT in contrast to patients relapsing while still on CT (90% vs 20%, P = 0.0170, Fig. 6). The three of seven patients with hematologic relapse during CT who got salvage CT showed no improvement of remission status, indicating the general refractory nature of the disease to chemotherapy here.

Three patients were treated with hypomethylating agents (HMA; decitabine or azacytidine) for relapse. Two patients showed stable disease for more than 100 days prior to alloHSCT. One patient progressed and finally had AD at alloHSCT. Two patients received HMA and Venetoclax (HMAClax) with one of them showing MRD+ stable disease until alloHSCT was performed 130 days later. The other one with hematological relapse achieved partial remission at alloHSCT 50 days later.

Fludarabine, amsacrine and cytarabine (FLAMSA, n = 22) based sequential conditioning, starting in median 49 days after relapse (range 1–78 days), did not improve outcome in patients with MRD+ CR or AD at transplant (statistical data not shown, Fig. 1).

### Relapse post alloHSCT

**Figure 7:**
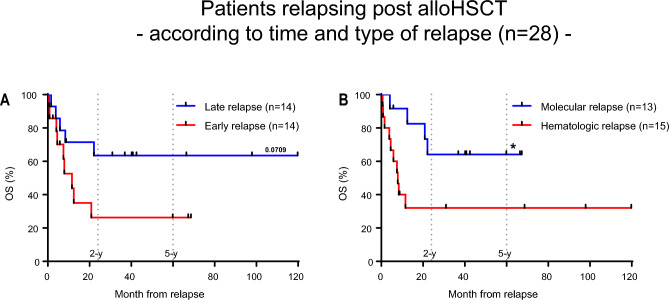
Kaplan–Meier survival curves. (**A**) Overall survival month from relapse after alloHSCT of all patients according to time of relapse. (**B**) Overall survival month from relapse after alloHSCT of all patients according to type of relapse. Log-rank (Mantel-Cox) test was used to test for statistical significance in survival curves. Statistical significance was established at asterisks displaying P values: *P < 0.05.

Of the 64 patients who received alloHSCT, 28 (44%) experienced either molecular (n = 13, 46%) or morphological (n = 15, 54%) relapse after a median of 118 days (range 28–1017 days) post alloHSCT (Fig. 7A). The 2-y-OS was 63% when relapse occurred after median relapse time of 118 days and 26% when relapse occurred earlier. Relapse detected on a molecular level was associated with significant better 2-y-OS than hematologic relapse post alloHSCT with 64% and 32% (P = 0.034), respectively (Fig. 7B).

Relapse was treated with combined therapeutic approaches including tapering off immunosuppression (n = 21), HMA (n = 22), donor lymphocyte infusion (n = 16), tyrosine kinase inhibitors (n = 7), HMAClax (n = 4), salvage CT (n = 7) and 2nd alloHSCT (n = 5). Fourteen patients (50%) achieved an ongoing molecular remission again, whereas 14 patients (50%) died from disease progression. GvHD was associated with long term disease control after relapse (data not shown).

## Discussion

In our cohort of patients with NPM^mut^ AML who require alloHSCT patients with MRD− CR at transplant had better outcomes than patients with MRD+ CR or patients with AD at transplant. In patients where the transplant indication was triggered by relapse or persistent disease post or during CT, outcome after alloHSCT was best when relapse was detected on a molecular level after completion of CT, demonstrating the importance of continuous MRD−monitoring via high sensitive RT-PCR. Patients with molecular or hematologic relapse after completing CT responded well to salvage CT in contrast to patients relapsing while still on CT. Patients who achieved a 2nd MRD− CR pre alloHSCT had a very good chance for long-term remission after alloHSCT. Patients with hematologic relapse during CT had poor response to salvage CT and did not achieve MRD− CR before transplant. Fludarabine, amsacrine and cytarabine (FLAMSA) based sequential conditioning did not improve outcome in patients with MRD+ CR or AD at transplant. Late relapses (defined as relapse occurred after median relapse time of 118 days) or relapses detected on a molecular level after alloHSCT is associated with a good chance of still achieving a long-term disease control after relapse, which explains the divergence between OS and PFS in the analyses shown here. Effective relapse strategies are available here, with particular focus on the occurrence of mild or moderate graft versus host disease (GvHD) probably reflecting an immunological graft versus leukemia (GvL) effect.

Dillon et al. showed in their study with 107 patients, that factors like high levels of MRD (above 200 copies per 10^5^ ABL in the PB or 1000 copies in the BM) and the presence of a FLT3-ITD mutation at diagnosis were associated with adverse outcome^16^. Kayser et al. identified the same threshold of 1000 copies per 10^5^ ABL in the bone marrow in their study of 39 patients with NPM1-mutated AML^15^. Finally, Bill et al. reported a significant difference in outcome according to the molecular MRD status in 51 patients with a lower threshold equivalent to 10 copies per 10^5^ ABL according to the technical characteristics of the digital droplet PCR platform employed^24^. In contrast to the aforementioned studies, we could not define a stringent MRD cut-off value, because as part of clinical routine we had results from different laboratories, who modified their high sensitive RT-PCR methods over the years.

Nevertheless, unlike these studies we were able to define two prognostic subgroups in the 2nd line group by time of relapse and response to salvage-CT. In our study, 10 of 15 patients (67%) with hematological or molecular relapse responded to salvage CT with 9 of 10 patients (90%) responding when relapse occurred after completing front-line conventional chemotherapy. Six of these patients (60%) achieved a 2nd MRD− CR and had an excellent outcome after alloHSCT. These results confirm data, reported by Dillon et al., where response to salvage therapy was associated with favorable survival after alloHSCT^16^. In contrast, only 1 of 5 patients (20%) relapsing during front-line therapy responded to salvage therapy, showing an unmet medical need for clinical studies with new drugs in these patients^25^. Here, the direct use of hypomethylating agents combined with Venetoclax may also be an effective alternative, especially because NPM1^mut^ AML (especially in combination with an IDH2-Mutation) reflects and specifically sensitive subgroup for this therapy^26^. Tiong et al. also showed the clinical impact of NPM1^mut^ molecular persistence after front line conventional chemotherapy^27^. Patients with NPM1^mut^ MRD positivity after completing front-line intensive chemotherapy had a variable course, with a substantial fraction (42%) remaining relapse-free at 1 year and 30% achieving MRD negativity. Preemptive salvage therapy prior to morphologic relapse, resulted in a significantly prolonged relapse free survival compared to those not receiving preemptive therapy^27^. Since in our cohort molecular relapse was defined by a dynamic MRD increase in two consecutive samples detected by RT-PCR, we did not assume the option of spontaneous MRD clearance, but impending hematologic relapse in these patients. The selection of conditioning regimens particularly for patients with AD or MRD positive CR remains controversial, and studies provide conflicting results^16,19,22,28–30^. Some studies suggest that myeloablative or sequential conditioning regimens should be preferred in patients who are MRD positive at transplant. As our data is retrospective and non-randomized, we could not see any additional benefit of a FLAMSA based sequential or myeloablative conditioning in these patients. Further improvement of current conditioning regimes may include the use of new agents like Venetoclax during conditioning rather than increasing dose intensity^31^.

Although the best data continue to be available for the effect of chemotherapy and hypomethylating agents combined with Venetoclax in patients with NPM1^mut^ AML, targeted therapies may also play a role in the future, particularly in the refractory situation. These are based on the strong expression of HOX genes in NPM1^mut^ AML, which is directly promoted by NPM1c through interaction with chromatin-bound proteins, including MLL1 and its cofactor Menin^32,33^. Menin inhibitors have already found their way into clinical studies, where clinical efficacy with tolerable toxicity has been demonstrated^34,35^. It would be advantageous in the future to be able to apply a maintenance therapy similar to the one used for patients with FLT3 mutations. However, the exact role remains to be demonstrated in phase II and III studies.

In patients with late or molecular relapses after alloHSCT effective relapse treatments were available and accompanying occurrence of controlled GvHD was associated with long-term disease control. Concomitantly, Dillon et al. observed a strong association between the use of T-depletion and adverse outcome of alloHSCT in patients with NPM1^mut^ AML^16^. These observations argue for less intensive post-transplant immunosuppression and immune interventions like preemptive donor lymphocyte infusions in the absence of GvHD in high-risk patients with MRD+ CR or even AD at transplantation.

To decrease the rate of failure of conventional chemotherapy in NPM1^mut^ AML the identification of additional risk factors and the impact of common concomitant mutations like DNMT3A mutations is crucial^36^. Since the data from our patients regarding a NGS panel that goes beyond cytogenetics and detection of NPM1 and FLT3 mutations, are incomplete, we did not want to discuss this further in our cohort.

Controversially to the ELN 2017 classification, the presence of unfavorable genetic alterations or mutations highly specific for secondary AML in the good risk group, identifies patients with a higher risk for relapse. These individuals could be seen as prime candidates for alloHSCT in 1st CR in the future^8,37^. Therefore, detailed complementary next generation sequencing analysis in addition to conventional standard PCR and cytogenetic analysis should always be performed. In addition, the recently published ELN 2022 guidelines must also be taken into account in the future, in which an additional FLT3-ITD already represents an intermediate risk, regardless of the ratio, which is reasoned here on the basis of the technical aspects of the determination of the ratio^2^.

In conclusion, according to our data outcome post alloHSCT in NPM1^mut^ AML depends on pre-transplant remission and molecular MRD status. Later relapses respond well to salvage CT and patients who achieve a 2nd MRD− CR before transplant have excellent long term survival. Furthermore, survival is superior if relapse is diagnosed as molecular relapse, which underlines the importance of intensive MRD monitoring. Patients who experience hematologic relapse during therapy represent a clinical challenge as they rarely achieve remission with conventional chemotherapy. Studies evaluating therapy augmentations such as the additional application of Venetoclax during salvage therapy or conditioning may be considered in the future.

## Supplementary Information

Supplementary Figure 1.

Supplementary Figure 2.

Supplementary Legends.

Supplementary Table 1.

Supplementary Table 2.

## Data Availability

Publication-related data are available from the corresponding author on reasonable request.
