# Supplementary material for: Allogeneic hematopoietic stem cell transplantation and pre-transplant strategies in patients with NPM1-mutated acute myeloid leukemia: a single center experience
Source: Sci Rep. 2023 Jul 4;13:10774. doi: 10.1038/s41598-023-38037-5 (PMC10319811; doi:10.1038/s41598-023-38037-5)

Figure S1

Patients receiving alloHSCT as 1st line therapy
because of additional genetic risk factors
- according to minimal residual disease before alloHSCT (n=25)

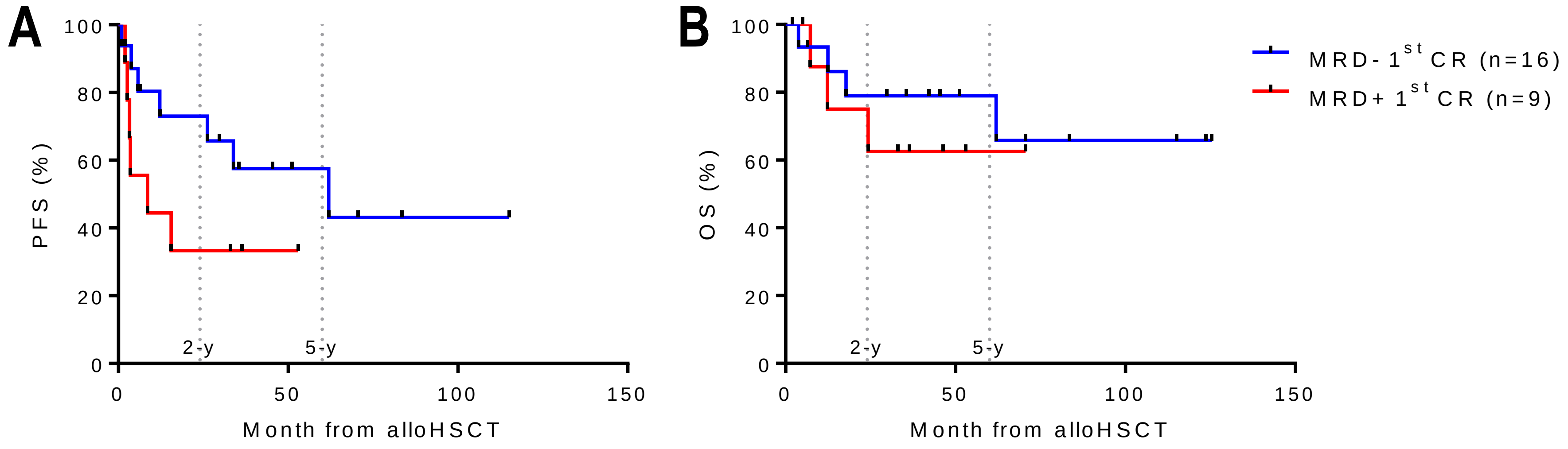

Supplement: Supplementary file 1 — Supplementary Figure 1. [file 41598_2023_38037_MOESM1_ESM.pdf]
