# Supplementary material for: Allogeneic hematopoietic stem cell transplantation and pre-transplant strategies in patients with NPM1-mutated acute myeloid leukemia: a single center experience
Source: Sci Rep. 2023 Jul 4;13:10774. doi: 10.1038/s41598-023-38037-5 (PMC10319811; doi:10.1038/s41598-023-38037-5)

Figure S2

Patients receiving alloHSCT as 1st line therapy in 1st CR
compared to patients with 2nd line indication at relapse
- according according to disease activity at relapse -

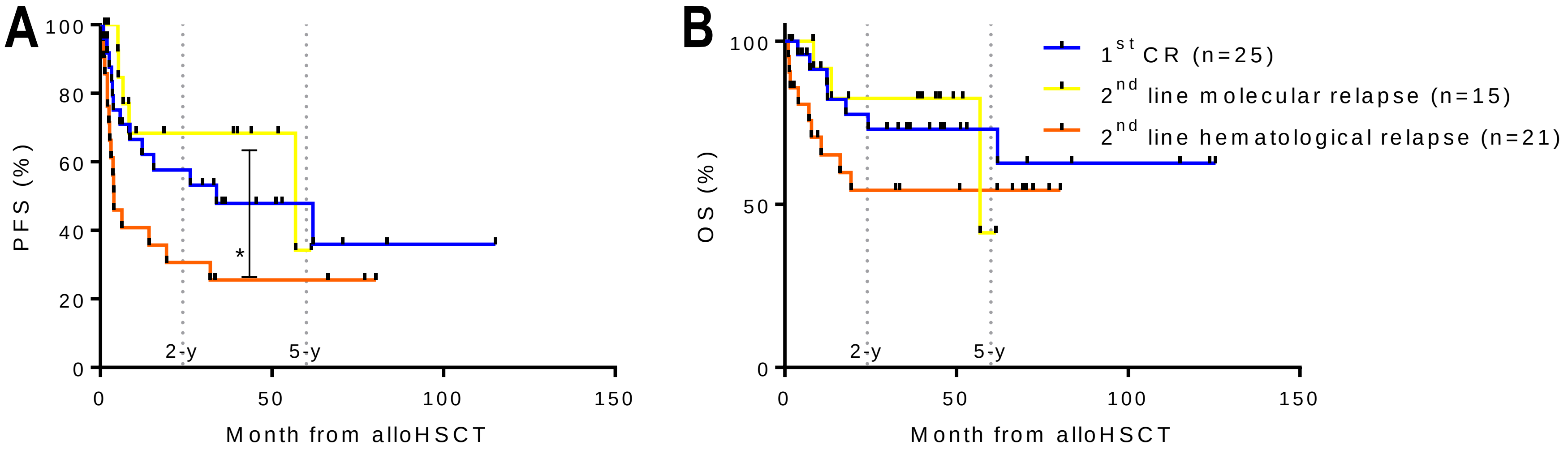

Supplement: Supplementary file 2 — Supplementary Figure 2. [file 41598_2023_38037_MOESM2_ESM.pdf]
