# Supplementary material for: Allogeneic hematopoietic stem cell transplantation and pre-transplant strategies in patients with NPM1-mutated acute myeloid leukemia: a single center experience
Source: Sci Rep. 2023 Jul 4;13:10774. doi: 10.1038/s41598-023-38037-5 (PMC10319811; doi:10.1038/s41598-023-38037-5)
Supplement: Supplementary file 3 — Supplementary Legends. [file 41598_2023_38037_MOESM3_ESM.pdf]

Figure legends supplement

Figure S1: Kaplan-Meier survival curves. (A) Progression free survival (PFS) and (B) overall survival (OS) month from alloHSCT of patients with 1st line indication according to pre alloHSCT MRD status. Log-rank (Mantel-Cox) test was used to test for statistical significance in survival curves.

Figure S2: Kaplan-Meier survival curves. (A) Overall survival and (B) progression free survival month from alloHSCT of patients with 1st line indication in comparison to patients with 2nd line indication according to relapse characteristics. Log-rank (Mantel-Cox) test was used to test for statistical significance in survival curves. Statistical significance was established at asterisks displaying P-values: *P < 0.05

Supplemental text to Figure 1: Twenty-seven (42%) patients had an indication for alloHSCT as first line therapy in first hematological remission. Two (3%) of them relapsed before alloHSCT and alloHSCT was performed with AD. Among the remaining 25 (39%) patients, 16 (25%) patients received alloHSCT in 1st MRD- CR and 9 (14%) patients in 1st MRD+ CR. The OS for patients who were transplanted in 1st CR was 75% with a relapse frequency of 31% for MRD- patients vs 67% with relapse frequency of 67% for patients with MRD+ CR. Thirty-seven (58%) patients received alloHSCT as 2nd line therapy. Fourteen (22%) patients relapsed or had inadequate response during CT and 23 (36%) patients relapsed post CT. Fifteen (23%) patients got salvage CT. The OS for 7 (11%) MRD- patients who were transplanted as 2nd line therapy was 86% without the occurrence of any relapse. One patient died of complications in the post alloHSCT course with ongoing MRD- CR. Fifteen (23%) patients with 2nd line indication were MRD+ pre alloHSCT and the OS was 67% with a relapse frequency of 33% (OS 50% and relapse frequency of 20% for the relapse

during CT group and OS 78% and relapse frequency of 44% for the relapse post CT group). Two patients died in relapse and 3 patients died of complications in the post alloHSCT course with ongoing MRD- CR. Fourteen (22%) patients had AD pre alloHSCT and the OS was 57% with a relapse frequency of 71%. (OS 43% and relapse frequency of 71% for the relapse during CT group and OS 71% and relapse frequency of 71% for the relapse post CT group). Five patients died in relapse and 1 patient died of complications in the post alloHSCT course with ongoing MRD- CR. One Patient with extramedullary relapse post therapy was MRD- in bone marrow, got radiatio and got alloHSCT with ongoing MRD- CR.
