# Supplementary material for: Allogeneic hematopoietic stem cell transplantation and pre-transplant strategies in patients with NPM1-mutated acute myeloid leukemia: a single center experience
Source: Sci Rep. 2023 Jul 4;13:10774. doi: 10.1038/s41598-023-38037-5 (PMC10319811; doi:10.1038/s41598-023-38037-5)
Supplement: Supplementary file 4 — Supplementary Table 1. [file 41598_2023_38037_MOESM4_ESM.pdf]

Table S1

Table S1. Therapy pre alloHSCT for patients with 1st line indication

Induction	Remission post induction	Additional induction	Consolidation	Additional treatment	Remission pre alloHSCT
1-2 # 7+3 or ICE	CR	–	0-3 # AraC 1-3g/m^2	Midostaurin 6 pts	13 pts MRD- CR
1 # 7+3	PR	1 # HAM	–	–	3 pts MRD- CR
1-2 # 7+3 or ICE	CR	–	0-2 # AraC 1-3g/m^2	Midostaurin 2 pts	7 pts MRD+ CR
1 # 7+3	CR	–	1 # HAM	–	1 pts MRD+ CR
1 # 7+3	No response	1 # HAM	–	–	1 pts MRD+ CR
1 # ICE	CR	–	1 # AraC 1g/m^2	–	1 pts AD
2 # 7+3	CR	–	3 # HAM	–	1 pts AD

Abbreviations: #: cycles; 7+3: anthracycline, days 1-3; cytosinarabinosid 200mg/m^2 /day in continuous infusion, days 1-7;. ICE: idarubicin 10mg/m^2/day, days 1-3; cytosinarabinosid 100mg/m^2 /day in continuous infusion, days 1-7; etoposide 100mg/m^2/day, days 1-5; CR: complete remession; PR: partial remission; HAM AraC 1-3g/m^2 every 12 hours, days 1-3, mitoxantrone 12mg/m^2, days 1-3; MRD: minimal residual disease positive (+) or negative (-); AD active disease
