# Supplementary material for: Allogeneic hematopoietic stem cell transplantation and pre-transplant strategies in patients with NPM1-mutated acute myeloid leukemia: a single center experience
Source: Sci Rep. 2023 Jul 4;13:10774. doi: 10.1038/s41598-023-38037-5 (PMC10319811; doi:10.1038/s41598-023-38037-5)
Supplement: Supplementary file 5 — Supplementary Table 2. [file 41598_2023_38037_MOESM5_ESM.pdf]

Table S2

Table S2. Therapy pre relapse and pre salvage strategy for patients with 2nd line indication					
Induction	Remission post induction	Additional induction	Consolidation	Additional treatment	No.
Relapse post front-line chemotherapy					
1-2 # 7+3 or ICE	CR	–	2-4 # AraC 1-3g/m^2	GO 6 pts	22 pts
1 # 7+3	PR	1 # HAM	3 # AraC 3g/m^2	–	1 pts
Relapse during front-line chemotherapy					
1-2 # 7+3 or ICE	CR	–	1-4 # AraC 1-3g/m^2	GO 3 pts	10 pts
1 # 7+3 or ICE	PR	1 -2 # HAM	1-3 # AraC 1-3g/m^2	–	2 pts
2 # 7+3	PR	–	–	GO 1 pts	2 pts

Abbreviations: Abbreviations: #: cycles; 7+3: anthracycline, days 1-3; cytosinarabinosid 200mg/m2 /day in continuous infusion, days 1-7;. ICE: idarubicin 10mg/m2/day, days 1-3; cytosinarabinosid 100mg/m2 /day in continuous infusion, days 1-7; etoposide 100mg/m2/day, days 1-5; CR: complete remession; PR: partial remission; HAM AraC 1-3g/m2 every 12 hours, days 1-3, mitoxantrone 12mg/m2, days 1-3; MRD: minimal residual disease positive (+) or negative (-); AD active disease; GO: Gemtuzumab Ozogamicin
